# Physical health examination in outpatients with schizophrenia: the cost effectiveness of laboratory screening tests

**DOI:** 10.1186/s12991-020-00321-3

**Published:** 2020-12-11

**Authors:** Saana Eskelinen, Janne V. J. Suvisaari, Jaana M. Suvisaari

**Affiliations:** 1 Psychiatry, University of Helsinki and Helsinki University Hospital, P.O. Box 590, 00029 Helsinki, Finland; 2 Department of Public Health Solutions, Mental Health Unit, National Institute for Health and Welfare, P.O. Box 30, 00271 Helsinki, Finland; 3 HUSLAB Laboratories, University of Helsinki and Helsinki University Hospital, P.O. Box 720, 00029 Helsinki, Finland

**Keywords:** Schizophrenia, Physical, Somatic, Screening, Laboratory

## Abstract

**Background:**

Guidelines on laboratory screening in schizophrenia recommend annual monitoring of fasting lipids and glucose. The utility and the cost effectiveness of more extensive laboratory screening have not been studied.

**Methods:**

The Living Conditions and the Physical Health of Outpatients with Schizophrenia Study provided a comprehensive health examination, including a laboratory test panel for 275 participants. We calculated the prevalence of the results outside the reference range for each laboratory test, and estimated the cost effectiveness to find an aberrant test result using the number needed to screen to find one abnormal result (NNSAR) and the direct cost spent to find one abnormal result (DCSAR, NNSAR x direct cost per test) formulas. In addition, we studied whether patients who were obese or used clozapine had more often abnormal results.

**Results:**

A half of the sample had 25-hydroxyvitamin D below, and almost one-fourth cholesterol, triglycerides or glucose above the reference range. One-fifth had sodium below and gamma glutamyltransferase above the reference range. NNSAR was highest for potassium (137) and lowest for 25-hydroxyvitamin D (2). DCSAR was below 5€ for glucose, all lipids and sodium, and below 10€ for creatinine and gamma glutamyltransferase. Potassium (130€), pH-adjusted ionized calcium (33 €) and thyroid stimulating hormone (33€) had highest DCSARs. Several abnormal results were more common in obese and clozapine using patients.

**Conclusions:**

An annual laboratory screening panel for an outpatient with schizophrenia should include fasting glucose, lipids, sodium, creatinine, a liver function test and complete blood count, and preferably 25-hydroxyvitamin D.

## Background

Health care workers providing services for people with schizophrenia and other severe mental disorders are advised to pay close attention to the physical well-being of their patients [1]. Patients with schizophrenia are prone to a variety of physical illnesses and risk factors, and have a shortened life-expectancy [2–4]. Especially cardiovascular diseases and metabolic disturbances, such as metabolic syndrome, type 2 diabetes and obesity, are substantially more common than in the general population [5–8].

Guidelines on laboratory monitoring in schizophrenia usually recommend fasting lipids and glucose testing annually after the first year of antipsychotic medication use [9]. The aim of the testing is to reveal increased cardiovascular risk, established diabetes, or severe hyperlipidemia, and subsequently offer preventive measures and/or timely treatment for these disturbances [1, 9]. In addition to the aforementioned laboratory tests, weight, blood pressure and cigarette smoking should be assessed at least once a year. Moreover, the British guideline for people with psychosis and schizophrenia recommends comprehensive, annual health checks that focus on common physical health problems [10]. Due to the extensive physical comorbidity [2], the question arises whether additional laboratory tests beyond fasting lipids and glucose that ought to be monitored in the annual physical health check for individuals with schizophrenia.

The need of laboratory screening among psychiatric patients has gained some scientific interest in the 1970s and 1980s [11, 12]. More recent research, conducted especially in the field of the emergency medicine, has mainly focused on the utility of medical clearance, i.e., use of laboratory testing in the differential diagnostics between physical and mental illnesses, once a patient with psychiatric symptoms is treated in the emergency department [13]. In addition, a few studies have examined thyroid function testing among psychiatric patients [14–16].

To our knowledge, three studies have assessed the need and the cost effectiveness of routine laboratory screening upon patient´s admission to a psychiatric facility [17–19]. Nowadays the treatment of schizophrenia is primarily provided on an outpatient basis. However, no studies exist on utility and cost effectiveness of routine laboratory screening panels as a part of physical health monitoring in outpatients with schizophrenia.

The aims of this study were to examine: (1) the proportion of test results outside the reference range in a basic laboratory panel and (2) the cost effectiveness of these tests among outpatients with schizophrenia. In addition, we studied whether patients who used clozapine or olanzapine or were obese had more abnormal laboratory results and would need more extensive laboratory screening.

## Methods

### The sample

The Living Conditions and the Physical Health of Outpatients with Schizophrenia Study offered a comprehensive health examination to patients with schizophrenia spectrum disorders treated in the outpatient clinic of Kellokoski Hospital, Finland, between 2009 and 2013. The participants filled in a questionnaire, attended an appointment by a nurse, had laboratory tests taken and attended an appointment with a general practitioner (GP). The GP checked patients´ medication use, took the medical history and conducted a structured clinical examination. The findings of the health examination were discussed and a written summary mailed to the patient and his/her GP. For the detailed description of the health examination protocol, see [3].

The study was approved by the Ethics Committee of the Hospital District of Helsinki and Uusimaa and by the Hyvinkää Hospital Area. All participants gave a written informed consent.

### Laboratory tests

The nurse instructed the patients verbally and with a written note to fast the night before the laboratory tests. The following fasting laboratory tests were included in the protocol: complete blood count with neutrophil count (CBC + Ne), plasma sodium, potassium, creatinine, glucose, total cholesterol, high (HDL) and low (LDL) density lipoprotein cholesterol, triglycerides, gamma glutamyltransferase (GGT), thyroid stimulating hormone (TSH), 25-hydroxyvitamin D (Vit D), and serum pH-adjusted ionized calcium (Ca-ion). The blood tests were taken in the morning and analyzed at HUS Helsinki University Hospital, HUSLAB Laboratories (HUSLAB).

### Calculations

The prevalence of results outside the reference range was calculated for each laboratory test. The costs of the laboratory tests in 2018 were obtained from HUSLAB laboratories. The expenses to find an aberrant test result were estimated in Euros using the NNSAR number needed to screen to find one abnormal result (NNSAR) and DCSAR direct cost spent to find one abnormal result (DCSAR) formulas as proposed by Arce-Gordon [18] and Lopez-Castroman [19]. NNSAR is calculated as 1/prevalence of abnormal results in the studied population, and DCSAR as NNSAR x direct cost per test [19].

## Results

Of 409 patients treated at the clinic, 275 (67.2%) participated in the health examination. The mean age of the participants was 44.9 years (SD 12.6) and 55.1% were men, 190 (68.8%) were diagnosed with schizophrenia, 49 (17.8%) with schizoaffective disorder, and 37 (13.4%) with other schizophrenia spectrum disorders (hereafter referred as schizophrenia). Of the sample, 96.4% used antipsychotic medication. Somatic medication use was common, as only 38 (13.8%) did not use any somatic drugs. Mean number of separate somatic medications was 3.3 (SD 2.69, min 0-max 13). Of the patients, 75 (27.2%) used lipid-lowering medication, 59 (21.4%) antihypertensives, 37 (13.4%) diabetes medication and 34 (12.3%) medication for hypothyroidism. A detailed description of the sample is given in Eskelinen et al. [3].

### Abnormal laboratory results

The frequency of laboratory results outside the reference range is presented in Table 1. The highest prevalence of aberrant results was in Vit D, where 129 (47.1%) of the participants had a level below 50 mU/l (low) and 8 (2.9%) below 20 mU/l (very low). Regarding metabolic test results, almost one-fourth of the participants had total cholesterol, triglycerides or glucose above the reference range. While 119 (43.4%) had LDL cholesterol above the reference range, 85 (31.0%) had low HDL cholesterol. Hyponatremia was common, 55 (20.1%) had sodium level below the reference range. GGT levels above the reference range were also relatively common (47 (17.2%)).

**Table 1:** Frequency of laboratory results that were below or above the reference range in 275 outpatients with schizophrenia

Laboratory test	Reference range, units	Frequency (*N* (%)) below the reference range	Frequency (*N* (%)) above the reference range	Mean (SD)	Min–max
B-Leukocytes^a^	3.4‒8.2, E9/l	4 (1.5%)	50 (18.3%)	6.7 (2.3)	2.1‒21.2
B-Neutrophils^b^	1.5‒6.7, E9/l	5 (1.8%)	17 (6.3%)	3.8 (1.9)	0.8‒19.3
B-Erythrocytes^a^	Men 4.25‒5.7 Women 3.9‒5.2, E12/l	9 (3.3%)	8 (2.9%)	4.8 (0.4)	3.0‒6.0
B-Thrombocytes^b^	150–360, E9/l	10 (3.7%)	16 (5.9%)	248.0 (65.0)	50‒464
B-Hemoglobin^a^	Men 134‒167 Women 117‒155, g/l	13 (4.7%)	10 (3.7%)	142.8 (14.2)	57‒175
B-Hematocrit^a^	Men 39‒50, Women 35‒46, %	6 (2.2%)	5 (1.8%)	42.7 (3.6)	21‒52
e-Mean corpuscular volume^a^	82‒98, fl	13 (4.7%)	2 (0.7%)	88.4 (4.2)	70‒100
e-Mean corpuscular hemoglobin^a^	27‒33, pg/cell	13 (4.7%)	0	29.6 (1.7)	19‒33
P-Potassium^a^	3.3‒4.9, mmol/l	0	2 (0.7%)	4.0 (0.3)	3.4‒5.1
P-Sodium^a^	137‒145, mmol/l	55 (20.1%)	2 (0.7%)	138.4 (3.2)	120‒147
S-pH-adjusted ionized calcium^c^	1.16‒1.3, mmol/l	24 (9.0%)	8 (3.0%)	1.2 (0.04)	1.03‒1.42
P-Creatinine^b^	Men 60‒100/women 50‒90, µmol/l	19 (7.0%)	16 (5.9%)	73.2 (18.0)	38‒237
P-Gamma glutamyltransferase^a^	Men < 60/women < 40, U/l	NCS	47 (17.2%)	38.7 (31.4)	10‒203
fP-Total cholesterol^a^	< 5, mmol/l	NCS	103 (37.6%)	4.8 (1.0)	2.4‒8.2
fP- Low density cholesterol^a^	< 3, mmol/l	NCS	119 (43.4%)	2.9 (0.9)	0.9‒5.8
fP- High density cholesterol^a^	Men > 1/ Women > 1.2, mmol/l	85 (31.0%)	NCS	1.4 (0.5)	0.48‒4.1
fP-Triglycerides^a^	< 1.7, mmol/l		104 (38.0%)	1.7 (1.1)	0.4‒7.0
fP-Glucose^a^	4.0‒6.0, nmol/l	0	106 (38.7%)	6.1 (1.1)	4.5‒11.2
P- Thyroid stimulating hormone^a^	0.5‒3.6, mu/l	7 (2.6%)	28 (10.3%)	2.1 (1.2)	0.3‒7.1
P- 25-Hydroxyvitamin D^a^	> 50, nmol/l	129 (47.1%)	NCS	53.1 (19.6)	11–124

^a^ Missing from one participant, ^b^ missing from two participants, ^c^ missing from eight participants

B, blood, P, plasma, S, serum, e, erythrocyte, f, fasting, SD, standard deviation, min, minimum value, max, maximum value

NCS, not clinically significant; only values above the reference range were taken into account in NNSAR

### Abnormal laboratory results related to clozapine or olanzapine medication and obesity

Clozapine was the most commonly used antipsychotic drug in the sample, 114 (41.5%) participants used it. When testing whether aberrant laboratory results differed between patients using clozapine and those using other antipsychotics, the following statistically significant results were found. Clozapine users had more often high triglycerides (47.8% vs. 31.1%, *p* = 0.005) and high glucose (46.0% vs. 33.5%, *p* = 0.037). Regarding the CBC + Ne, clozapine users had more often low thrombocyte count (8.0% vs. 0.6%, *p* = 0.002). Instead, mean corpuscular hemoglobin (0.9% vs. 7.5%, *p* = 0.009) and mean corpuscular hemoglobin concentration (2.7% vs. 11.8%, *p* = 0.004) were less often below the range. Erythrocyte count (0% vs. 5.0%, *p* = 0.013) was less often high. In addition, clozapine users had less often low sodium (10.6% vs. 26.7%, *p* = 0.001) and high TSH (4.4% vs. 14.4%, *p* = 0.008). As a secondary analysis, we compared patients who used olanzapine to the other patients, excluding those who used clozapine. The only significant differences were that patients who used olanzapine more often had high lymphocyte count (15.0% vs. 4.2%, *p* = 0.030) and high creatinine (17.5% vs. 2.5%, *p* = 0.003).

Obesity was common: 47.6% of participants had a body mass index (BMI) above 30. Several abnormal laboratory results were more common in obese patients: HDL cholesterol was more often low (41.1% vs. 21.8%, *p* < 0.001), whereas glucose (49.6% vs. 28.9%, *p* < 0.001), triglycerides (55.8% vs. 21.8%, *p* < 0.001), GGT (28.7% vs. 7.0%, *p* < 0.001) and TSH (15.5% vs. 5.0%, *p* = 0.004) were more often above the reference range. In CBC + Ne, low lymphocyte count (8.8% vs. 18.4%, *p* = 0.023), and low neutrophil count (0% vs. 3.5%, *p* = 0.040) were less common, and high erythrocyte count (6.2% vs. 0%, *p* = 0.002) more common.

### Cost-effectiveness of laboratory testing

The number needed to screen to find one abnormal result (NNSAR) was highest for potassium (137) and lowest for Vit D [2]. Direct cost spent to find one abnormal result (DCSAR) was highly variable: below 5€ for glucose, all lipids and sodium, and below 10€ for creatinine and GGT. DCSARs were highest for potassium (130 €), Ca-ion (33 €) and TSH (33€). Calculating DCSAR was complex for the CBC + Ne. The total cost for the test was used for each of its components, which overestimates the cost of one test. Of the patients, 111 had any abnormal test result in the CBC + Ne, based on this the DCSAR would be 7.4€. The costs of the separate tests in 2018 in HUSLAB, and their NNSSARs and DCSARs are presented in Table 2.

**Table 2:** Costs, NNSAR and DCSAR of laboratory tests

Laboratory test	Cost^d^	NNSAR^a^	DCSAR^b^
B-CBC + Ne^c^	3.0 €	2.469	7.4 €
P-Potassium	0.95 €	137	130.2 €
P-Sodium	0.95 €	4.8	4.6 €
S- pH-adjusted ionized calcium	4.0 €	8.3	33.4 €
P- Creatinine	0.95 €	7.8	7.4 €
P- Gamma glutamyltransferase	0.95 €	5.8	5.5 €
fP-Total cholesterol	1.10 €	2.6	2.9 €
fP- Low density cholesterol	1.30 €	2.3	3.0 €
fP- High density cholesterol	1.30 €	3.2	4.2 €
fP-Triglycerides	1.10 €	2.6	2.9 €
fP-Glucose	0.95 €	2.6	2.5 €
P- Thyroid stimulating hormone	4.20 €	7.8	32.9 €
P-25-Hydroxyvitamin D	7.0 €	2.1	14.9 €

^a^NNSAR, number needed to screen to find one abnormal result (1/prevalence of abnormal result)

^b^DCSAR, NNSAR × direct cost

^c^Complete blood count with neutrophil count

^d^In 2018 HUS Helsinki University Hospital HUSLAB Laboratories

B, blood, P, plasma, S, serum, f, fasting

## Discussion

We evaluated the proportion and the cost-effectiveness of abnormal results in a basic laboratory test panel in a sample of outpatients with schizophrenia. Most participants of our study had a long duration of psychotic disorder and were clinically stable, and the majority of them had medications for physical illnesses and multiple physical health care needs [3].

If the direct cost spent to find one abnormal result (DCSAR) is used as the criterion to select screening laboratory tests, our results support the inclusion of glucose, lipids, sodium, creatinine, GGT, and CBC in the protocol. The DCSAR was below 10 € for all of them, and from 13 to 43% of the patients had abnormal test results in these tests. In an earlier study of newly hospitalized psychiatric patients, of whom 39% had a schizophrenia spectrum disorder, abnormal test results were less common, except for total cholesterol [18]. However, DCSAR values used in the study by Arce-Cordon are not directly comparable with ours, as we used the costs from the laboratory serving in our catchment area.

If the test with the highest number of abnormal results (NNSR) was to be chosen for screening, Vit D ought to be routinely measured. Vit D deficiency has been shown to be more common among individuals with psychosis compared to the general population [20, 21], and it increases the risk of osteoporosis and fractures, common in this patient group [22–23]. In Finland a systematic Vit D fortification of liquid dairy products and fat spreads was started in 2003, and the dose was doubled in 2010. As a result, the mean Vit D concentration has increased at the population level from 48 (2000) to 65 (2011) [24]. Among the participants of our study conducted between 2009–2013, the mean Vit D level was lower (53). In addition, Vit D deficiency (< 50) was five times as common, compared to the Finnish adult population in 2011: 47% vs. 9% [24]. To date, the evidence does not support screening for Vit D deficiency in community-dwelling, asymptomatic adults [25]. However, patients with schizophrenia could be considered as a high-risk population for whom screening would be indicated. They often have an unhealthy diet and stay indoors, are smokers, have difficulties in moving, and may have extrapyramidal, anticholinergic and hypogonadal side-effects from antipsychotic medication, features that increase the risk of Vit D deficiency, falls and fractures. The DSCAR of Vit D testing was 15€ because the test is fairly expensive compared to the other tests. However, the testing could be reasonable due to straight forwardness of prescribing a Vit D supplementation for a deficient patient.

One in ten of the sample had TSH above the reference range (at highest 7.1), indicative of subclinical hypothyroidism. Among psychiatric patients, reversible and irrelevant abnormalities in thyroid function tests are frequent and often due to other factors such as acute psychiatric state, starvation or substance use, rather than to a thyroid problem per se [14]. Lachman and Garnier do not recommend TSH testing upon admission to psychiatric hospital due to low clinical utility and high costs [16]. In our analysis, TSH had the second highest DCSAR of all tests (33 €) and eight patients had to be screened to get an abnormal result. Interestingly, elevated TSH was three times more common in obese than in non-obese patients. The relationship between thyroid function and obesity has been reported to be bidirectional: hypothyroidism is associated with weight gain, but obesity also influences thyroid function [26]. The association between elevated TSH and obesity is mainly regarded as an adaptation, and the appropriate therapy is adjustment of energy balance and body weight [27]. Based on the current evidence, an annual screening of TSH in obese patients with schizophrenia is not necessary, but the possibility of hypothyroidism should be kept in mind in patients with rapid weight gain.

Elevated GGT was strongly associated with obesity in our sample. This finding, along with a notable proportion of obese patients with high triglycerides and low HDL cholesterol would fit with the features of non-alcoholic fatty liver disease (NAFLD) [28]. Obesity is the major risk factor for NAFLD, and a subtype of NAFLD (NASH, non-alcoholic steatohepatitis) may progress to liver cirrhosis, cancer and extrahepatic diseases [29, 30]. In a study assessing first episode, drug-naïve psychosis patients, one-fourth with normal NAFLD index at the baseline had an index indicative of NAFLD at 3-year follow-up from the initiation of antipsychotic medication [31]. Several risk factors of liver disease are common in patients with schizophrenia: obesity, medications involving hepatic metabolic pathways, higher prevalence of hepatitis C and alcohol use disorder. Therefore, we recommend routine monitoring of a liver function test among patients with schizophrenia.

Clozapine, along with olanzapine, is metabolically the most hazardous antipsychotic drug [32], yet the most effective one [33]. Our findings are in line with the previous research: patients using clozapine hade more often triglycerides and glucose above the reference range. It should be noted that hypertriglyceridemia can be dangerous. Drug-induced acute pancreatitis may rarely be secondary to antipsychotic use-induced hypertriglyceridemia (usually triglyceride level has to rise above 10) [34]. In our sample, thrombocytes were more often below the reference range among clozapine users. However, clozapine-induced thrombocytopenia is often mild and transient and seldom causes clozapine discontinuation [35].

As a secondary analysis, we excluded patients who used clozapine and compared patients who used olanzapine to the other patients. There were no differences in lipids or glucose levels. However, olanzapine is often switched to another antipsychotic if significant weight gain or metabolic abnormalities emerge [36], and therefore current users may be those who were less prone to these side-effects. Regarding elevated creatinine in patients using olanzapine, a recent study found that second-generation antipsychotic use was associated with elevated risk of chronic kidney disease, and the highest risk was found for clozapine use, followed by olanzapine [37]. As there were only 41 patients who used olanzapine in our study sample, this finding warrants further investigation in a larger study sample.

In the 1980s study of 205 stable psychiatric outpatients, with mixture of psychiatric diagnoses, 43% had high fasting glucose, 14% anemia and 5% hyponatremia [11]. In our study almost 40% had high glucose, less than 5% low hemoglobin and 20% hyponatremia. In the study by Beresford [11], anemia was especially prevalent among the aged participants, whereas our study group consisted mainly of middle-aged patients. Higher prevalence of hyponatremia may be due to common use (32% of the sample) of antidepressive medication, mainly selective serotonin reuptake inhibitors, which may cause hyponatremia [32]. However, primary polydipsia is also relatively common in patients with schizophrenia and may contribute to the high prevalence of hyponatremia as well [38].

According to a recent review, a routine laboratory screening is not recommended for patients admitted in the emergency department for a psychiatric reason [13]. In the outpatient care, the rationale for laboratory screening is somewhat different. Patients often develop metabolic disturbances during the treatment with antipsychotics, and the use of other psychotropics (e.g., lithium, valproic acid, antidepressants) may require laboratory monitoring as well [32]. In addition, concomitant use of psychiatric and somatic medications may cause need for laboratory controls (e.g., selective serotonin reuptake inhibitors and diuretic medication may increase the risk of hyponatremia).

### Clinical implications

Routine screening without intervening, when needed, will not improve the health of patients with schizophrenia, instead it leads to extra expenses and workload. Psychiatrists ordering the laboratory test panels may be unaware if an abnormal result has a clinical significance*.* Hence, they should be assisted in the interpretation of the laboratory test results and medical decision-making by straightforward algorithms (e.g., Lester tool, SCORE) and by straightforward consultations by a GP or a somatic specialist. In some countries, primary health care services are advised to organize regular health evaluations for patients with schizophrenia. In these settings, close collaboration between primary care practitioners and psychiatrists is needed, because some of the abnormal test results may be related to psychotropic medication or symptoms like polydipsia. Moreover, since metabolic disturbances (glucose, lipids, liver function tests) are extremely common among patients with schizophrenia, targeted prevention and treatment of obesity should be emphasized at all levels of the health care system, from the onset of the psychotic disorder.

Of outmost importance is to ensure that the patients receive and understand the laboratory test results. Being aware that, e.g., the glucose level is above the reference range may motivate the patient to lose weight and increase physical activity and seek professional help for the condition if needed. For example, in the case of Vit D, patients would probably adhere better to use the vitamin supplementation if they are aware of their vitamin level.

### Strengths and limitations

Our study sample can be considered representative of Finnish outpatients with schizophrenia. All outpatients with schizophrenia from three municipalities—one urban and two rural—were invited to the study, and the participation rate was good. Clozapine was used by over 40 percent of the sample, consistently with the high prevalence of clozapine use in Finland [39]. Therefore, we were also able to study abnormal test results found in patients using clozapine compared to patients using other antipsychotics.

A limitation of the study is the original choice of laboratory tests. It covered tests we assumed to be reasonable in a routine health evaluation but could have been more comprehensive. In addition, we used GGT as a liver function test instead of alanine aminotransferase (ALT). ALT could have been more reasonable choice since it is more specific for liver damage, whereas GGT has high sensitivity but low specificity [40].

Furthermore, 32.2% patients treated in the clinic refused to participate, which might have caused self-selection bias [3]. We have no health information from the non-participants, but there were no age or gender differences between participants and non-participants [3].

This was a cross-sectional, observational study. The next step would be to carry out longitudinal studies to examine the long-term effectiveness of routine health evaluations in the reduction of morbidity and mortality in patients with schizophrenia.

## Conclusions

According to our results, glucose, lipids, sodium, creatinine, liver function test, CBC, and preferably Vit D ought to be screened annually among outpatients with schizophrenia. There is no need for more extensive routine laboratory monitoring among clozapine users or obese patients, with the exception of TSH if the weight gain has been rapid. Most importantly, clinically significant abnormal laboratory test results should be evaluated and followed by relevant interventions.

## Data Availability

Data are from the Living Conditions and the Physical Health of Outpatients with Schizophrenia Study, whose authors may be contacted at University of Helsinki and Helsinki University Hospital, (principal investigator: Saana Eskelinen, saana.eskelinen@hus.fi). In research collaboration, sharing of the data is possible but requires amendment to the ethics committee permission and a separate agreement with University of Helsinki and Helsinki University Hospital. The ethics committee will evaluate on a case-by-case basis whether the intended collaboration is concordant with the consent given by the participants.

## Abbreviations

NNSAR: Number needed to screen to find one abnormal result
DCSAR: Direct cost spent to find one abnormal result
GP: General practitioner
CBC + Ne: Complete blood count with neutrophil count
HDL: High density lipoprotein cholesterol
LDL: Low density lipoprotein cholesterol
GGT: Gamma glutamyltransferase
TSH: Thyroid stimulating hormone
Vit D: 25-Hydroxyvitamin D
Ca-ion: Serum pH-adjusted ionized calcium
HUSLAB: HUS Helsinki University Hospital, HUSLAB Laboratories
NAFLD: Non-alcoholic fatty liver disease
NASH: Non-alcoholic steatohepatitis
ALT: Alanine aminotransferase
